# Electro-acupuncture promotes survival, differentiation of the bone marrow mesenchymal stem cells as well as functional recovery in the spinal cord-transected rats

**DOI:** 10.1186/1471-2202-10-35

**Published:** 2009-04-20

**Authors:** Ying Ding, Qing Yan, Jing-Wen Ruan, Yan-Qing Zhang, Wen-Jie Li, Yu-Jiao Zhang, Yan Li, Hongxin Dong, Yuan-Shan Zeng

**Affiliations:** 1 Division of Neuroscience, Department of Histology and Embryology, 74# Zhongshan 2nd Road, Guangzhou, PR China; 2 Center for Stem Cell Biology and Tissue Engineering, 74# Zhongshan 2nd Road, Guangzhou, PR China; 3 Institute of Spinal Cord Injury, Zhongshan School of Medicine, Sun Yat-sen University, 74# Zhongshan 2nd Road, Guangzhou, PR China; 4 Department of Acupuncture of the first Affiliated Hospital, Sun Yat-sen University, Guangzhou, PR China; 5 Department of Psychiatry and Behavioral Sciences, Northwestern University, Feinberg School of Medicine, 303 East Chicago Avenue, Chicago Illinois 60611-3008, USA

## Abstract

**Background:**

Bone marrow mesenchymal stem cells (MSCs) are one of the potential tools for treatment of the spinal cord injury; however, the survival and differentiation of MSCs in an injured spinal cord still need to be improved. In the present study, we investigated whether *Governor Vessel *electro-acupuncture (EA) could efficiently promote bone marrow mesenchymal stem cells (MSCs) survival and differentiation, axonal regeneration and finally, functional recovery in the transected spinal cord.

**Results:**

The spinal cords of adult Sprague-Dawley (SD) rats were completely transected at T10, five experimental groups were performed: 1. sham operated control (Sham-control); 2. operated control (Op-control); 3. electro-acupuncture treatment (EA); 4. MSCs transplantation (MSCs); and 5. MSCs transplantation combined with electro-acupuncture (MSCs+EA). After 2-8 weeks of MSCs transplantation plus EA treatment, we found that the neurotrophin-3 (NT-3), cAMP level, the differentiation of MSCs, the 5-HT positive and CGRP positive nerve fibers in the lesion site and nearby tissue of injured spinal cord were significantly increased in the MSCs+EA group as compared to the group of the MSCs transplantation or the EA treated alone. Furthermore, behavioral test and spinal cord evoked potentials detection demonstrated a significantly functional recovery in the MSCs +EA group.

**Conclusion:**

These results suggest that EA treatment may promote grafted MSCs survival and differentiation; MSCs transplantation combined with EA treatment could promote axonal regeneration and partial locomotor functional recovery in the transected spinal cord in rats and indicate a promising avenue of treatment of spinal cord injury.

## Background

Functional deficits following spinal cord injury (SCI) result from neuronal damage, demyelination of axons, and dysfunctional neuroglial cells. The failure of axonal regeneration following SCI has been attributed to a non-permissive environment containing inflammatory mediators, lack of neurotrophic support, and inhibitory molecules. Numerous therapeutic strategies attempt to overcome these negative factors and promote axon regeneration, including transplanting tissue bridges [1,2], artificial scaffolds [3,4], cellular transplants of various cells types [5-11], growth factors [12,13], and various combinatorial treatments [14-18]. However, at present, there still is a lack of effective treatments for spinal cord injuries.

Previously, we reported that co-implanting Schwann cells and neural stem cells (NSCs) into a semi-transected or completely transected spinal cord significantly affected its recovery under certain circumstances [19,20]. However, neural stem cells face limitations inherent in their procurement from fetal tissue, including histocompatibility and ethical concerns for clinical use. Bone marrow mesenchymal stem cells (MSCs) have been suggested as an alterative in spinal cord injury research and a promising candidate for the treatment of central nervous system injuries [21,22]. Potential advantages of MSCs over other types of transplanted cells include their ability to be harvested from autologous donors, their relatively rapid expansion in vitro, and the possibility that allogeneic MSCs are not immunogenic and hence, readily available for clinical use [21]. MSCs also secrete a variety of growth factors and cytokines that could contribute to reparation after a CNS injury [23-25]. Studies have suggested that MSCs can differentiate into neural phenotypes [26-31], which would enable them to replace lost neural cells after a brain or spinal cord injury. However, the limitations of MSCs include their poor survival and differentiation into neurons when transplanted into injured spinal cord [22,32,33].

Acupuncture is a therapeutic technique used in traditional Chinese medicine. Since its development several thousand years ago, acupuncture has made many contributions to healthcare and medical treatment. Electro-acupuncture (EA) is a type of therapy in which a needle inserted into an acupoint is attached to a trace pulse current with the purpose of producing synthetic electric and needling stimulation. The application of EA for the treatment of SCI has shown promising results in the alleviation of the patients' suffering [34]. EA is used on *'Governor Vessel' *acupoints for the treatment of spinal cord injury because the impairment of this channel is regarded as the essence of spinal cord damage in traditional Chinese medicine. The use of EA on the *Governor Vessel *has been shown to alleviate secondary damage after a spinal cord injury in both patients and animal models [35,36]. Interestingly, our previous study found that utilizing EA on the *Governor Vessel *promotes NT-3 secretion in the injured spinal cord and enhances the differentiation of grafted neural stem cells (NSCs) into neuron-like cells in the injured site [37]. Furthermore, the combination of *Governor Vessel *EA and NSCs transplantation promotes axonal regeneration and improves function in completely transected spinal cords of rats [38,39]. In this study, we investigated whether the transplantation of MSCs, combined with EA treatment could stimulate and promote survival and differentiation of transplanted MSCs, axonal regeneration, and functional improvement of the injured spinal cord.

## Methods

### Preparation of bone marrow mesenchymal stem cells (MSCs)

MSCs were prepared from one-month-old Sprague-Dawley (SD) rats in accordance with the procedure described in detail in our previous study [31]. The tibias and femurs of the SD rats were dissected under anesthesia and aseptic conditions. After removing the end of the bone, 5 ml of Dulbecco's modified Eagle medium (DMEM, Gibco/BRL, Carlsbad, CA) was injected into the central canal of the bone to extrude the bone marrow. The solution containing bone marrow was then centrifuged at 1000 rpm for 5 min. The pellet was resuspended in DMEM and supplemented with 10% inactivated fetal bovine serum (FBS), penicillin (100 U/ml), and streptomycin (100 mg/ml). The cells were then cultured in a 75 ml cell flask and after plating the cells for 48 h, the medium was replaced to remove non-adherent cells. When the adherent MSCs grew to near confluency, they were serially passaged using 0.25% trypsin/0.02% EDTA. After being passaged 3–5 times, the MSCs were ready for use in transplantation.

To verify the survival and transdifferentiation of transplanted MSCs, third or fourth generation MSCs were labeled with bromodeoxyuridine (BrdU, 1 μmol/L, Sigma) and Bisbenzimide (Hoechst33342, 10 μg/1 ml, Sigma). Two days before transplantation, MSCs were treated with 1 μmol/L BrdU for 48 h. In addition, MSCs were labeled with blue nuclear fluorescence Hoechst 33342 (10 μg/1 ml) 2 h before transplantation. Next, the MSCs were washed 3 times with D-Hank's balanced salt solution (HBSS, pH 7.4), and removed from the culture flasks using 0.25% trypsin/0.02% EDTA (Sigma). MSCs were centrifuged and resuspended in DMEM medium for cell counting. After undergoing the trypan blue dye exclusion test, the cell suspension was adjusted to a final concentration of 1×10^5 ^viable cells/μl in culture medium for transplantation.

### Animal groups and spinal cord surgery

Experiments were performed on 66 adult female SD rats (Experimental Animal Center, Zhongshan School of Medicine of Sun Yat-sen University, China) weighing 230~250 g. Rats were housed in a temperature-controlled (24 ± 2°C) and light-controlled (12:12 light-dark cycle) room with free access to food and water. Prior to experimental manipulation, rats were allowed to acclimate to the housing facilities and were handled daily at least for 3 days. All experimental protocols and animal handling procedures were approved by the Animal Care and Use Committee of Sun Yat-sen University, and were consistent with the National Institutes of Health Guide for the Care and Use of Laboratory Animals.

The rats were divided into 5 groups. The first group, the sham-control group, received only a laminectomy (Sham-control). The remaining four groups underwent complete transections at the T10 spinal segment of the spinal cord as follows: the Op-control group received a spinal cord transection only; the EA group received EA treatment after the spinal cord transection; the MSCs group received MSCs transplantation after spinal cord transection; and the MSCs+EA group received transplanted MSCs with EA treatment after transection.

T9–T10 laminectomy: Briefly, the animals were anesthetized with 1% sodium pentobarbital (40 mg/kg, i.p.). A laminectomy was carried out at the T9-T10 level to expose the T10 spinal segment. The dura was cut and the T10 segment was transected completely with a scalpel with no tissue removed [40,41]. A complete transection of the spinal cord was verified histologically. In the Op-control and EA groups, a piece of gelfoam (2 × 2 × 2 mm^3^, preinjected DMEM 5 μl) was inserted into the lesion site. In the MSCs and MSCs+EA groups, a small piece of gelfoam, which was preinjected with a MSCs suspension (1 × 10^5 ^cells/μl, 5 μl), was placed into the transected site of the spinal cord. After surgery and gelfoam transplantion, the dura was sutured, and the muscle and skin were closed in layers. In the sham-control group, rats received a laminectomy without any further spinal cord damage. After surgery, all rats received an intramuscular injection of penicillin (160,000 U/ml/d) and were housed separately in individual cages with thick soft bedding. Manual emptying of the bladders was performed twice daily. In the EA and MSCs+EA groups, rats received EA treatment every other day, beginning the seventh day post-spinal cord transection for a total of 7 weeks.

### Electro-acupuncture treatment

**Figure 1:**
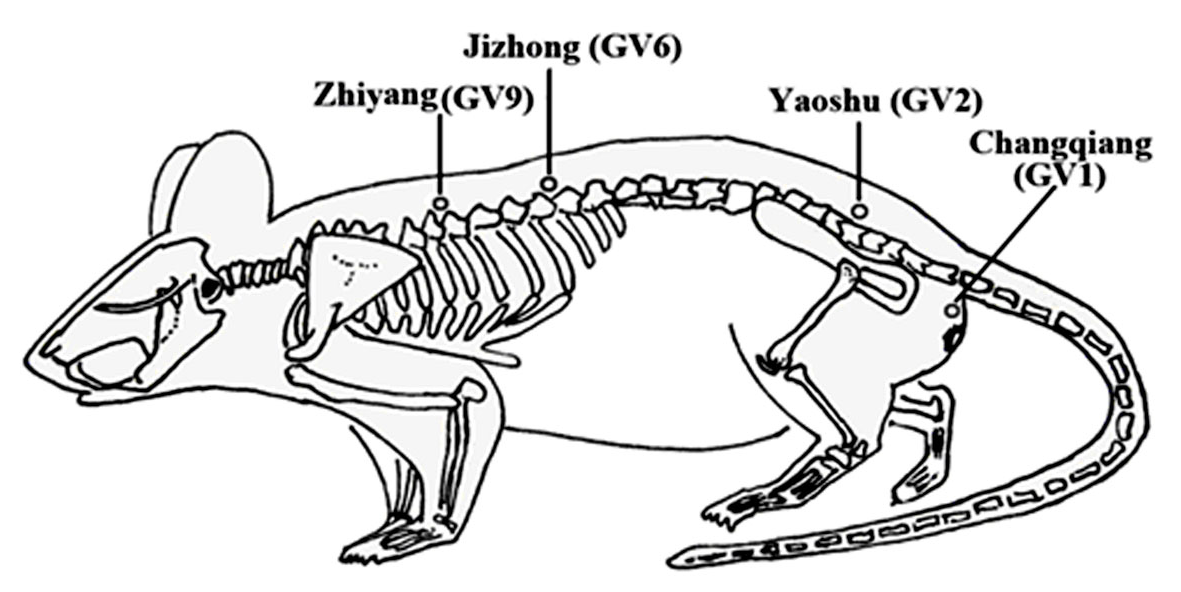
**Schematic diagram indicates the four selected acupoints of the 'Governor vessel'**. Changqiang (GV1), Yaoshu (GV2), Jizhong (GV6), and Zhiyang (GV9), which correspond to equivalent acupoints in humans.

Two pairs of '*Governor Vessel*' acupoints were utilized during EA treatment. One pair of acupoints is Changqiang (GV1) and Yaoshu (GV2); another pair of acupoints is Jizhong (GV6) and Zhiyang (GV9) (Fig. 1). The acupoint GV1 is located at the midpoint between the tip of the coccyx and the anus in prone position. GV2 is located on the posterior midline and in the hiatus of the sacrum in prone position. The acupoint GV6 is located on the posterior midline and in the depression below the spinous process of the eleventh thoracic vertebra in prone position. GV9 is located on the posterior midline and in the depression below the spinous process of the seventh thoracic vertebra in prone position. GV6 and GV9 are located on the depression below the rostral and caudal spinous process of transected spinal cord, respectively. EA on both GV6 and GV9 acupoints can directly treat the injured spinal cord. EA on both the GV1 and GV2 acupoints can improve emptying the bowels and bladder and lumbar vertebrae pain.

Rats were loosely immobilized in a specially made restrainer that exposed their dorsal spine, hind legs, and tails. Two pairs of stainless silver needles of 0.3 mm in diameter were inserted into the GV1 and GV2, and GV6 and GV9, to a depth of 5 mm. The two pairs of needles were connected to the output terminals of an electro-acupuncture apparatus (Model G 6805-2, Shanghai Medical Electronic Apparatus Company, China). Alternating strings of dense-sparse frequencies (60 Hz for 1.05 s and 2 Hz for 2.85 s) were used. The intensity was adjusted to induce a slight twitch of the hindlimb (≤ 1 mA) and lasted for 20 min. EA was administered once every other day for 7 weeks, starting from the seventh day post-surgery.

### cAMP and NT-3 assay

In accordance with our previous work [39], cAMP levels were detected 2 weeks after the spinal cord transection. Sixteen rats (N = 4 for each group) were anesthetized with 1% sodium pentobarbital (40 mg/kg) and transcardially perfused with 200 ml of ice-cold 0.1 M PB. Subsequently, a 1 cm long segment of T9–10 spinal cord containing the lesion site was quickly removed and dissected on dry ice. The segments were weighed and permeabilized with ice-cold ethanol. The extracts were lyophilized in a speed vacuum (SC110A, Savant Industries, Farmingdale, NY) and stored at -70°C until analysis. The acetylation protocol was used to increase the signal strength of cAMP [42]. The extracts were solubilized in assay buffer then diluted in a mixture of one part acetic anhydride to two parts triethylamine. A cyclic AMP binding compound, 3', 5'-cyclic phosphoric acid 2'-0-succinyl-3-[^125^I]-iodotyrosine methyl ester, was added to the solution, followed by the anti-cAMP antiserum. After incubation for 15~18 h at 4°C, Amerlex-M secondary antibody was added to the sample, and the precipitate was obtained by centrifugation. An autosampling gamma scintillation counter (Beckman, Fullerton, CA) counted each sample for 60 s and the cAMP values were obtained using a standard curve.

Previously, we tested the NT-3 level 2 weeks after the spinal cord transection and found the NT-3 level increased after EA treatment [37]. In the present study, we investigated whether EA treatment could maintain the higher level of NT-3 in the lesion site for a sustained period following the spinal cord injury. Therefore, we decided to detect the NT-3 level four weeks after the spinal cord transection. Four weeks after transection of the spinal cord, 19 rats (N = 4 for each group, except sham control = 3) were anesthetized with 1% sodium pentobarbital (40 mg/kg) and transcardially perfused with 200 ml of ice-cold 0.1 M PB. Three spinal cord segments, the lesion site and the areas 1 cm rostral and 1 cm caudal to the lesion site, were excised while on dry ice. The segments were weighed and then mechanically homogenized in ice-cold 0.1 M PB. Homogenates were centrifuged for 10 min at 14,000 rpm at 4°C and used for NT-3 ELISA according to the instructions of the manufacturer (NT-3 Emax ImmunoAssay System; Boster).

### Behavioral analysis

Both the BBB locomotor rating scale and inclined-grid climbing test were used to analyze the post-surgical motor behavior of the subjects' hindlimbs [20,43]. The first test quantitatively evaluates voluntary hindlimb movement and body weight support. The open field locomotor activity score was determined by observation and scoring of behaviors involving the trunk, tail, and hindlimbs. Each session lasted 4 minutes. Scores from both examiners were averaged for each rat. Scores ranged from 0 to 21 (0, no movement; 21, normal movement). The second test qualitatively assesses accuracy of foot placement and coordination, which differentiates local reflex activity from voluntary movement. All animals underwent behavioral testing every 2 weeks post-surgery for 8 weeks. The tests were videotaped and both examiners were blind to each group when they participated in behavioral evaluation.

### Spinal cord evoked potentials (SCEP)

8 weeks after the spinal cord surgery, 23 rats (N = 5 in each group except sham control group = 3) were anesthetized with ketamine (40 mg/kg) and 1% sodium pentobarbital (30 mg/kg) and stereotaxically fixed. T1-L1 vertebrae were completely exposed. Briefly, The stimulation electrode was inserted into the T1-T2 interspinous ligaments, and a pair of needle electrodes was inserted into the interspinous ligaments of T12 to L1 for SCEP recording, according to a previous study [44]. The electrodes were then connected to the BL-410E Data Acquisition Analysis System for Life Science (Taimeng, China). The Parameter Settings of the SCEP signal are as follows: gain parameter 2000, time constant 0.01s, filtering 300 Hz. To elicit a SCEP, a single pulse stimulation, 50 ms in duration at a frequency of 5.1 Hz and with a 1 mV voltage density, was transmitted through the electrodes. The voltage density was set so that it elicited a mild twitch of the animal's vertebral body. In order to obtain high-quality waveforms for the SCEP signals, 100 SCEP responses were averaged for each rat. After performing evoked potentials on all subjects, the spinal cords were retransected to investigate whether the evoked potentials vanished.

### Immunohistochemical staining

Following the behavioral testing and evoked potentials recording, 23 rats of 5 groups were perfused by 4% paraformaldehyde in phosphate buffer (pH 7.4). Spinal cords were removed, post-fixed overnight in the paraformaldehyde, and cryoprotected in 0.1 M PB containing 30% sucrose at 4°C. Longitudinal cryosections (25 μm thickness) of spinal cord were cut and mounted on gelatin-coated slides for immunostaining. Primary antibodies were used as follows: mouse anti-oligodendrocyte special protein (clone CE-1, alternate name: MOSP, 1:1000, Chemicon); mouse anti-ED1 (a mouse monoclonal IgG, 1:200, Serotec, Indianapolis, IN); mouse anti-postsynaptic density (95 KDA) protein (PSD-95, 1:200, Sigma); rabbit polyclonal anti-serotonin (5-HT, 1:200, Sigma); rabbit polyclonal anti-calcitonin gene-related peptide (CGRP, 1:8000, Chemicon); rabbit polyclonal anti-glial fibrillary acidic protein (GFAP, 1:80, Sigma); rabbit polyclonal anti-synapsin I (1:500, Chemicon); rabbit polyclonal anti-neurofilament 150 KD (NF150, 1:400, Chemicon); rabbit polyclonal anti-β-tubulin III (1:100, Sigma). Cy3-conjugated goat anti-mouse IgG and Cy3-conjugated rabbit anti-goat IgG (1:800, Jackson Immunoresearch Labs, Inc.), FITC-conjugated goat anti-mouse IgG and FITC-conjugated goat anti-rabbit IgG (1:200, Jackson Immunoresearch Labs, Inc.) were used as secondary antibodies. Sections were washed three times with PBS and incubated with 10% normal goat serum with 0.3% Triton X-100 in PBS for 30 min at room temperature. Incubations with appropriate primary antibodies were performed overnight at 4°C. After repeated washing with PBS, sections were incubated with their respective secondary antibodies for 1 h at 37°C, washed with PBS, coverslipped, and examined under the fluorescence microscope.

Immunofluorescence double-labeling was performed to assess the survival and differentiation of transplanted MSCs. BrdU-labeled transplanted MSCs were identified by a mouse anti-BrdU antibody and visualized with FITC-conjugated goat secondary antibodies. Rabbit polyclonal anti-NF150 (for neurons) or rabbit polyclonal anti-β-tubulin III (for newly differentiated neurons) were used to identify the surviving MSCs that had differentiated into neurons.

A surviving cell count was conducted in 10 longitudinal spinal cord sections dissecting each animal's injury site, as a preceding study reported [19]. The surviving MSCs (labeled by BrdU) were counted in 3 randomly selected unit areas (0.09 mm^2^), delineated by a calibrated reticle eyepiece, distributed throughout the lesion site of each section. The average number of BrdU-labeled cells from the unit areas was designated surviving cells and used for comparison. Differentiated neuron-like cells and oligodendrocyte-like cells were also counted in the unit areas and expressed as a percentage of total surviving engrafted cells for use in comparison analysis.

Quantitative analysis of 5-HT and CGRP positive nerve fibers was performed according to prior studies [17,45]. A calibrated reticle eyepiece was used to delineate regions 300 μm rostral to the transection site, at the transection site, and 300 μm caudal to the transection site (see Additional file 1: PDF1 for a sketch of the injured spinal cord used to clarify the location of quantification). The 5-HT-labeled nerve fiber profiles were quantified in all regions at 200 × magnification, while the CGRP-positive fibers were quantified only in the lesion site at 200 × magnification. The spinal cords were cut in longitudinal sections, and every 5th section was mounted on a gelatin-coated slide. Ten sections per rat were analyzed and the total number of labeled fibers in all regions of each experimental group was averaged. The 5-HT or CGRP-positive fibers longer than 50 μm were counted as positive fibers.

### Data analysis

The total surviving MSCs and the percentages of differentiated cells were compared statistically between the MSCs+EA and MSCs groups using the Independent Samples T test. Other data were analyzed using one-way ANOVA or repeated-measure ANOVA. If equal variances were found, Fisher's least significant difference test was performed. Otherwise, the Kruskal-Wallis Test and Dunnett's T3 were used. The statistical significance level was set at P < 0.05.

## Results

### cAMP and NT-3 level in transected spinal cord

**Figure 2:**
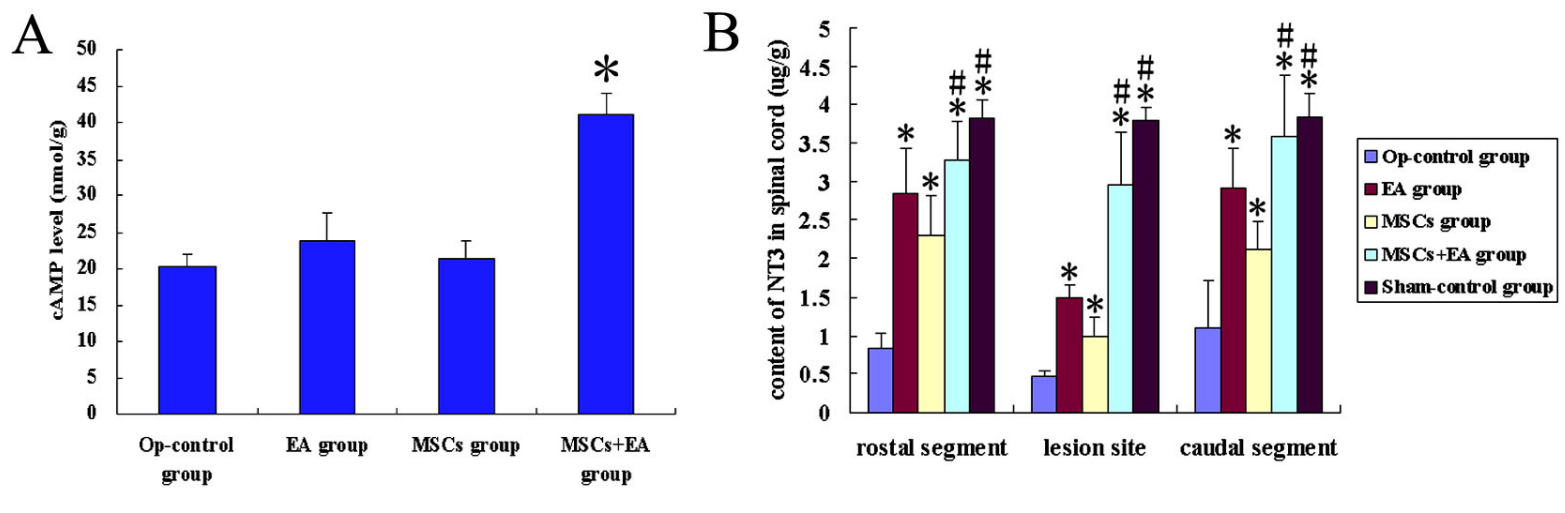
**cAMP and NT-3 level in the injured spinal cord**. A. cAMP levels in the lesion tissue were measured by radioimmunoassay. In the MSCs+EA group, cAMP levels in the lesioned tissue were significantly higher as compared to the other groups. *P < 0.001. Data = means ± SD. B. NT-3 contents in the segments rostrally and caudally to the lesion site, and the lesion site were measured by ELISA. In all three areas, the NT-3 contents were significantly increased in the EA, MSCs, and MSCs+EA groups as compared to the Op-control group (* indicates P < 0.01). In the lesion site, the NT-3 contents were significantly higher in the MSCs+EA group than the MSCs and EA groups (# indicates P < 0.01). Moreover, the NT-3 contents in the rostral and caudal segments to the lesion site in the MSCs+EA group had no significant difference as compared to the Sham-control group (P > 0.05). Data= means ± SD.

Two weeks after the spinal cord surgery, the cAMP levels in the lesion site of spinal tissue were significantly increased in the MSCs+EA group compared to other groups (F = 49.32, df = 3, P = 0.000), but there was no significant difference between the Op-control group, EA group, and MSCs group (Fig. 2A).

Four weeks following the spinal cord surgery, the NT-3 content at the lesion site in the MSCs+EA group was significantly increased compared to the MSCs or EA groups. However, NT-3 content at the rostral and caudal segments of the MSCs+EA group was not significantly different from that of the EA group. NT-3 in the Op-control group was the lowest (Fig. 2B). Our results indicate that transplanted MSCs and/or EA therapy may increase NT-3 level in the spinal tissue.

### Behavioral testing

**Figure 3:**
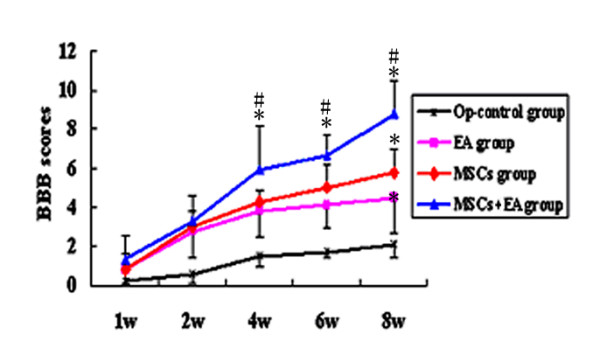
**BBB open field test: BBB scores were obtained starting from the first week throughout the 8 week survival period (5 time points)**. The scores gradually increased with time in each group. BBB scores were significantly higher in the three treatment groups as compared to that of the Op-control group from two weeks to eight weeks post-transection (P < 0.05). The MSCs+EA group showed a significantly higher score than the other treatment groups beginning the 4th week following transection. Especially at eight weeks after the operation, the scores of the MSCs+EA group were significantly higher than those of the other groups (*indicates P < 0.01 as compared to the Op-contorl group; # indicates P < 0.01 compared to the EA group/MSCs group). Data = means ± SD.

After spinal cord transection, all the rats were paralyzed and moved by pulling themselves forward with their forelimbs. Moreover, they presented with urinary and fecal incontinence. The hindlimb locomotor activity of rats in each group was evaluated by the BBB open field test at five time points between 1 and 8 weeks post-lesion. Hindlimb locomotor activity improved gradually in the three treatment groups throughout the entire follow-up survival period. BBB scores gradually increased over time in each group. BBB scores were significantly higher in the three treatment groups compared to the Op-control group in the period from two weeks to eight weeks post-lesion (P < 0.05). However, MSCs+EA group showed a significant higher score compared with the EA and MSCs groups after the fourth week, most notably at the eighth week following the operation (P < 0.01) (Fig. 3).

An alternative function test, the inclined grid test examining limb coordination, was also performed. Rats in the MSCs+EA group voluntarily placed their paws on the grid and pushed themselves upwards using their hindlimbs to reach the horizontal platform. Rats in the MSCs and EA groups placed their paws on the grid occasionally. In contrast, rats in the SCI group struggled to climb onto the inclined grid with their forelimbs while their hindlimbs dragged passively behind their body and their hind paws often fell through the mesh. Thus, this test showed voluntary movement of the hindlimbs and plantar placement primarily in the MSCs+EA group.

### Spinal cord evoked potentials (SCEP)

**Figure 4:**
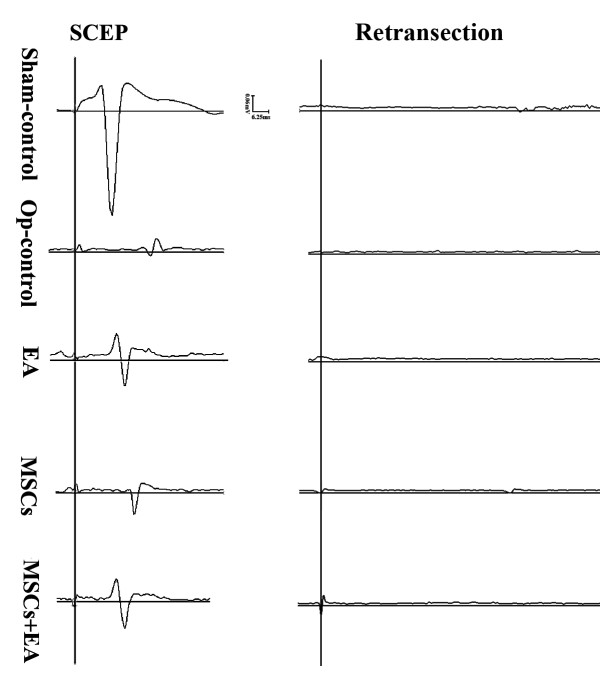
**Spinal cord evoked potential recovery after MSC transplantation and EA treatment**. A. The SCEPs of the rats in the Op-control group were very weak. After receiving EA, MSCs, or the combination therapy of EA and MSCs grafts, the SCEP of the rats recovered to different degrees. B. The restored activity of SCEP was abolished by a retransection, indicating descending axonal regeneration.

**Figure 5:**
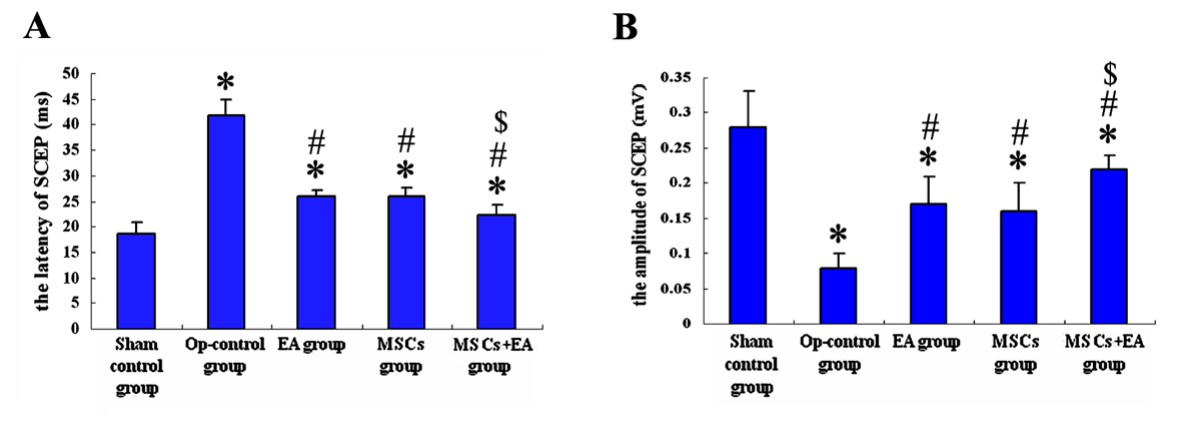
**The latency and amplitude of SCEP at the end of 8 weeks after MSCs transplantation and EA treatment**. A. After spinal cord transection surgery, the latency of SCEP was significantly prolonged in Op-control group as compared with the Sham-control group (* indicates P < 0.05). However, the MSCs+EA group showed a significant recovery of the latency (# indicates P < 0.01, as compared to the Op-control group; $ indicates P < 0.05, as compared to the EA/MSCs group). B. After spinal cord transection surgery, the amplitude of SCEP was significantly declined in the Op-control group as compared to the Sham-control group (* indicates P < 0.05). However, in the MSCs, EA, or MSCs+EA group, the amplitude of SCEP was significantly recovered as compared with the Op-control group (# indicates P < 0.05). The MSCs+EA group showed a significant recovery of the amplitude as compared to the EA or MSCs group ($ indicates P < 0.05). Data = means ± SD.

At the end of the eighth week following spinal cord injury, the SCEP in the Op-control group was weak, the latencies were prolonged, and the amplitudes were significantly decreased as compared with the other four groups (Fig. 4A, Fig. 5). However, the latencies of SCEP were significantly shorter (Fig. 5A) and the amplitudes were increased (Fig. 5B) in the EA+MSCs group compared to the MSCs or EA group alone. Representative sample traces show that SCEP stimulation normally evokes a short-latency positive-negative-positive wave, which is absent below an acute transection of the spinal cord, and is partially restored by EA, MSCs, or their combined treatment. Notably, this restored activity is abolished by a retransection, which indicates descending axonal regeneration (Fig. 4B).

### Survival and differentiation of MSCs in transected spinal cord

**Figure 6:**
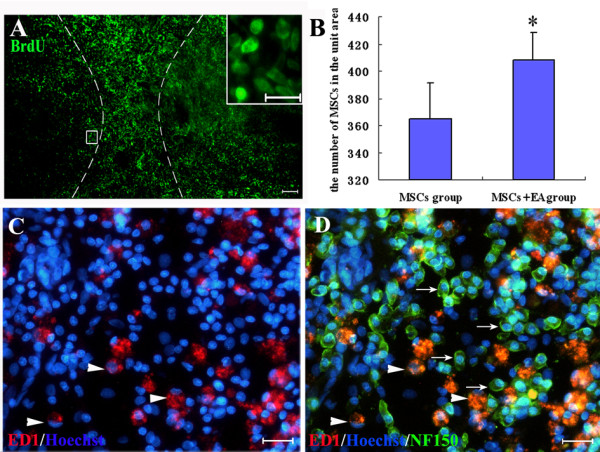
**The survival and differentiation of MSCs in the injured spinal cord**. A. BrdU-labeled nuclei (green) of MSCs in the injured spinal cord in the MSCs+EA group. Grafted BrdU-labeled MSCs were well integrated with host tissue and migrated into host tissue over the host/lesion interface in the spinal cord both rostrally and caudally. Two broken lines outline the lesion site. Inset in A showing the BrdU-labeled MSCs – at a higher magnification (Scale bars: 80 μm; Insets, 20 μm.). B. The number of survived MSCs in the lesion site (MSCs+EA vs. MSCs, * indicates P < 0.05). Data = means ± SD. C. ED1-immunostained activated macrophages (red) in the lesion site; numerous grafted MSCs (Hoechst labeled the nuclei of MSCs, blue) survived in the lesion site, some were phagocytosed by ED1 immunoreactive activated macrophages (arrowheads). D. NF150-positive neuron-like cells (green, arrows) co-localized with Hoechst-labeled nuclei of MSCs (Hoechst, blue), but not with ED1-positive activated macrophages (red). Scale bars: A = 80 μm, C and D = 20 μm.

During the 8th week after transplantation, the grafted cells labeled by BrdU could easily be identified with fluorescent microscopy, were mainly found in the lesion site, and were well integrated with the host tissue (Fig. 6A). These cells migrated rostrally and caudally into host tissue over the host/lesion interface in the spinal cord. Furthermore, statistical analyses showed that the number of surviving MSCs in the MSCs+EA group was significantly higher than that of the MSCs group (t = 2.861, df = 8, P < 0.05, Fig. 6B). Although most Hoechst-labeled MSCs seemed to be clearly intact in outline, some were fragmented and phagocytosed by ED1 immunoreactive activated macrophages (Fig. 6C). The ED1-labeled macrophages were still present at the lesion site, the margins around the lesion, and the lesion cavity in all groups, which indicated that there was still inflammatory action in the injured spinal cord 8 weeks after transection. A double-label immunofluorescence study showed that some NF150-positive neuron-like cells colocalized with the Hoechst-labeled nuclei of MSCs, but not with ED1-positive activated macrophages (Fig. 6D). The double-label immunostaining of NF-150 and ED1 indirectly proved the NF-150 positive cells in the graft site were from grafted MSCs, rather than non-specific staining or autofluorescence of activated macrophages.

**Figure 7:**
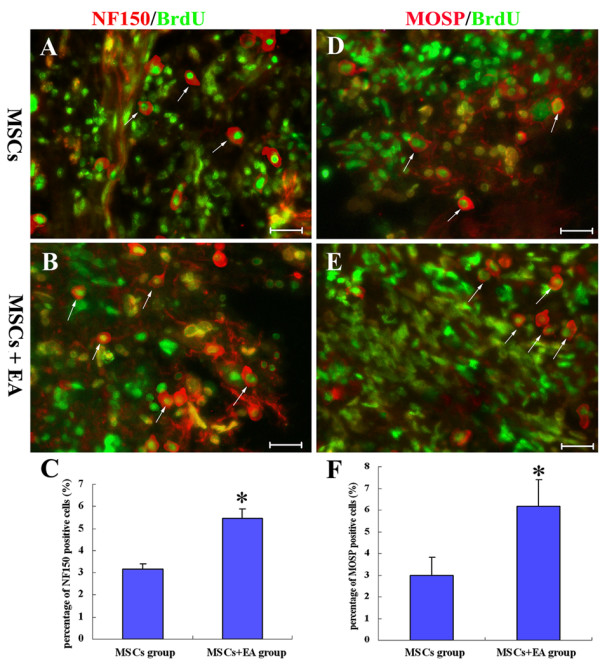
**Differentiation of MSCs into neuron-like and oligodendrocyte-like cells at 8 weeks following transplantation**. A and B: NF150 (red) and BrdU (green) double-labeling show the NF150-positive neuron-like cells (arrows) in the MSCs group (A) and MSCs+EA (B) group. Cell number counting indicates the number of NF150/BrdU double-labeled cells was significantly higher in the MSCs+EA group compared to the MSCs group (C, * indicates P < 0.05). D and E: MOSP (red) and BrdU (green) double-labeling show the MOSP-positive oligodendrocyte-like cells (arrows) in the MSCs group (D) and MSCs+EA group (E). Cell number counting indicates the number of MOSP/BrdU double-labeled cells was significantly higher in the MSCs+EA group as compared to the MSCs group (F, * indicates P < 0.05). Data= means ± SD. Scale bars: A, B, D, E = 20 μm.

The longitudinal spinal cord sections were examined for differentiation markers for neuron, oligodendrocyte, and astrocyte lineages. Fluorescent immunohistochemistry staining revealed that transplanted MSCs were able to differentiate into either neuron-like cells or glial-like cells. We found that the majority of differentiated neural cells of the engrafted MSCs were localized in the host/lesion interfaces and the lesion site. The transplanted cells partially expressed the neuronal marker, NF150 (Fig. 7A, B). Statistical analysis indicated significantly more neuron-like (NF150-positive) cells in the MSCs+EA group as compared with the MSCs group (t = -10.296, df = 8, P < 0.001, Fig. 7C). The percentage of differentiated neuron-like (NF150-positive) cells is 3.18% and 5.47% in the MSCs and MSCs+EA groups, respectively. The grafted cells partially expressed the oligodendrocyte marker, MOSP, which generally labels oligodendrocytes and central nerve myelin, but not peripheral nerve myelin (Fig. 7D, E). Statistical analysis showed a significant difference between the MSCs+EA (6.16%) group and the MSCs group (2.98%) (t = -4.658, df = 8, P < 0.01, Fig. 7F). Surprisingly, few GFAP-positive astrocytes colocalized with BrdU-labeled MSCs.

**Figure 8:**
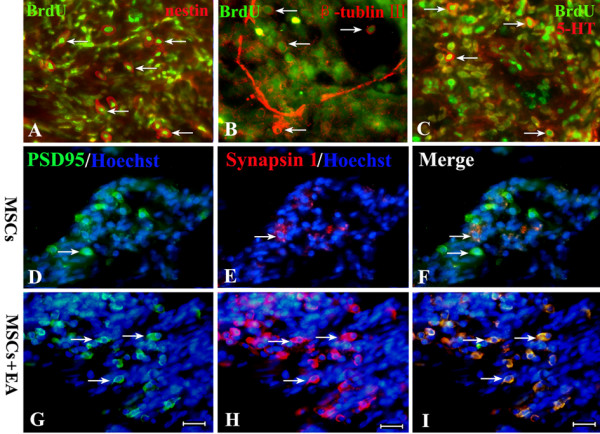
**Differentiation of MSCs into neuron-like cells at 8 weeks following transplantation**. A, B and C: Using different makers (nestin for neuroblast cells (A), β-tublin III for young neurons (B), and 5-HT for functional neurons (C) combined with a BrdU double-label), we found that MSCs transplanted into injured spinal cord tissue could survive and differentiate into neuronal phenotypes in the MSCs+EA group. D-F: In the MSCs group, a double immunofluorescence study of PSD95 (green) and synapsin I (red) showed a small number of coexpressing cells. G-I: Double immunofluorescence showed that some cells coexpressed PSD95 (green) and synapsin I (red) in the grafts of the EA+MSCs group. Scale bars: 20 μm.

At early stages of the transplantation process, many grafted cells expressed nestin, a marker for neuroblast cells (Fig. 8A). Then, some of the BrdU-labeled grafted cells expressed β-tublin III and NF150, markers of immature and mature neurons (Fig. 8B, Fig. 7A, B). Surprisingly, a few BrdU-labeled MSCs co-expressed 5-HT (Fig. 8C). Immunofluorescence double-labeling also showed some cells co-expressed PSD95 and synapsin I in the MSCs+EA group (Fig. 8G–I), but there were scant double-labeled grafted cells in the MSCs group (Fig. 8D–F). Since ≥ 95% of MSCs co-labeled with BrdU and Hoechst in vitro and in vivo (see Additional file 2: PDF2 for the BrdU and Hoechst co-labeling images in vitro and in vivo used to clarify the percentage of co-labeled with BrdU and Hoechst), we believe that Hoechst-labeled cells that co-express PSD95 and synapsin I are grafted cells. We also noticed that morphology of NF150, β-tubulin III, and 5-HT positive cells differentiated from grafted MSCs were round with few/no processes, which is not the typical morphology of neuron-like cells. Therefore, we think that these positive cells have neuronal phenotypes at least to some degree, but their structures are still immature.

### 5-HT and CGRP fibers regeneration

**Figure 9:**
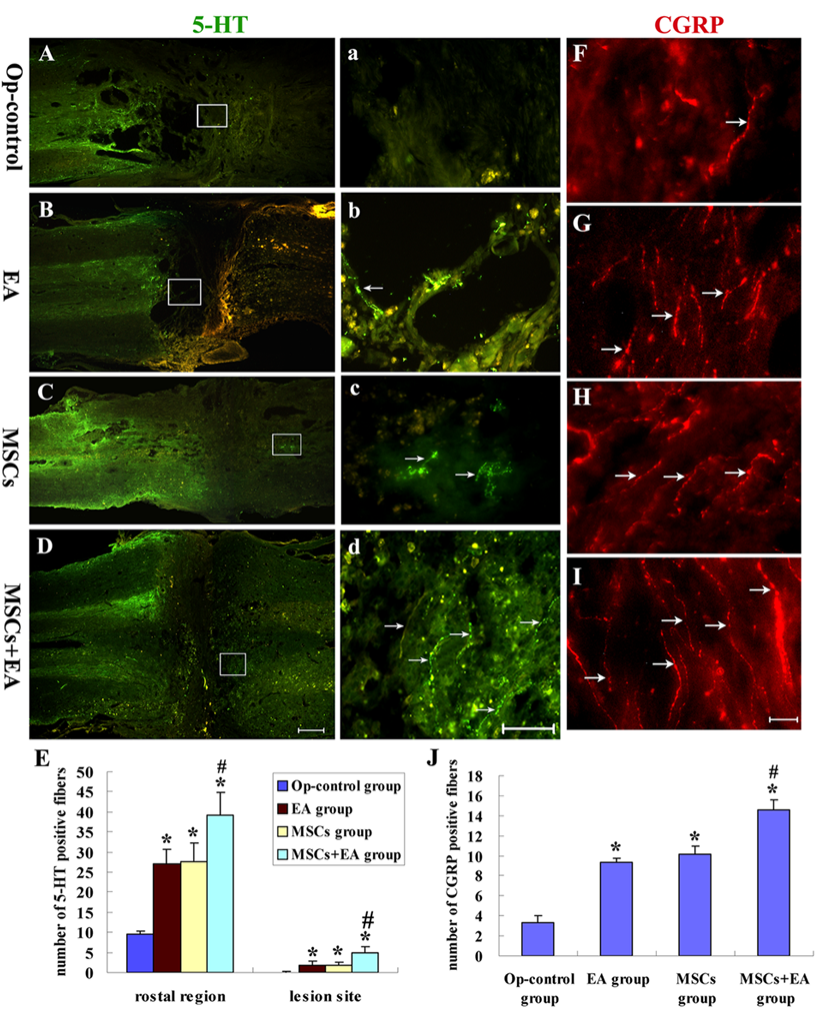
**5-HT and CGRP-positive nerve fibers in the injured spinal cord at 8 weeks following surgery**. A-D: A longitudinal section of spinal cord shows 5-HT positive fibers in the Op-control group (A), EA group (B), MSCs group (C), and MSCs+EA group (D). a-d: Higher magnification of the rectangle boxes in Fig. A-D. Scale bars: A, B, C, D = 320 μm; a, b, c, d = 40 μm. E: Comparison of the number of 5-HT positive fibers in the lesion site and the region immediately rostral to the lesion site among the 4 groups (MSCs+EA vs. Op-control group, * indicates P < 0.01; MSCs+EA vs. MSCs or EA groups: # indicates P < 0.05). F-I: A longitudinal section of spinal cord shows CGRP-positive fibers (arrows) in the Op-control group (F), EA group (G), MSCs group (H), and MSCs+EA group (I). Scale bars: F, G, H, I = 20 μm. J:Comparison of the number of CGRP-positive fibers located in the lesion site in the 4 groups (MSCs+EA vs. Op-control group, *indicates P < 0.001; MSCs+EA vs. EA or MSCs groups, # indicates P < 0.001).

Serotonergic fibers originate in brain stem nuclei, especially in the raphe nuclei, and project throughout the longitudinal extent of the spinal cord. These fibers are thought to have a greater growth capacity following spinal cord injury and can be conveniently detected by 5-HT immunostaining [11,18]. In the Op-control group, 5-HT-positive fibers grew up to the boundary of the lesion, but did not cross the host/lesion interface into the lesion site (Fig. 9A, a). In addition, 5-HT-positive fibers were present in the rostral host tissue near cysts in the vicinity of the central lesion site, and a few 5-HT-positive fibers passed along the margins of the cysts. A few 5-HT-positive fibers in the EA group crossed the rostral host/lesion interface into the lesion site (Fig. 9B, b). In the MSCs group, some 5-HT-positive fibers extended into the lesion site and one of the 5 subjects had 5-HT-positive fibers crossing the host/lesion interface into the host caudal cord. The most caudally located axons often exhibited tortuous projections, suggesting sprouting (Fig. 9C, c). Unexpectedly, 3 of the 7 MSCs+EA animals had visible 5-HT-positive fibers in the host tissue caudal to the lesion site (Fig. 9D, d), and the number of the fibers in the rostral spinal cord crossing the rostral host/lesion interface into the lesion site were dramatically increased in the MSCs+EA group (Fig. 9E). CGRP-positive fibers, which label local sensory axons, were also significantly increased in the lesion site of the MSCs+EA group compared to both the MSCs and EA groups (Fig. 9F–J).

## Discussion

In the present study, we evaluated the effect of EA on the survival and differentiation of MSC transplantation, axonal regeneration, as well as functional recovery in rats with transected spinal cords. We found that the level of cAMP and NT-3, the number of vital and differentiated MSCs, the number of 5-HT-positive and CGRP-positive fibers in and near the lesion site of the injured spinal cord were all significantly increased in the MSCs+EA group as compared to the groups that underwent MSC transplantation or EA treatment alone. Furthermore, evidence from BBB scales and spinal cord evoked potentials demonstrated a significant functional recovery in the MSCs +EA group.

MSCs transplantation combined with EA treatment increased the cAMP level in the lesion site of the spinal cord, which is consistent with our previous study [39]. The underlying mechanism of cAMP elevation is unclear. However, it is possible that the pulsed electric field of EA therapy could cause depolarization of neurons [46,47], which would cause Ca^2+ ^influx via L-type Ca^2+ ^channels, followed by Ca^2+^-induced elevation of intracellular cAMP levels via Ca^2+^/calmodulin-dependent adenylyl cyclase pathway [48]. Moreover, intracellular Ca^2+ ^elevation caused by neuronal depolarization may stimulate an autocrine neurotrophic mechanism, leading to the synthesis and release of neurotrophic factors, NT-3 and BDNF, by the neurons themselves [49,50]. Thus, EA may stimulate the depolarization of neurons, which causes the opening of certain voltage-gated ion channels of neuroglia cells, which subsequently stimulates a rise in the intracellular cAMP level and autocrine release of neurotrophic factors. cAMP elevation can increase recruitment of the TrkB receptor to the plasma membrane of retinal ganglion cells [51], suggesting that cAMP may promote neuronal survival by increasing neurotrophin receptor availability and signaling.

In previous studies, we found that EA treatment increases the amount of NT-3 in the spinal cord tissue surrounding the lesion site 2 weeks after spinal cord transection [37,39]. In addition, when studying the effects of MSC grafts combined with EA treatment on a spinal cord injury, we obtained similar results in which EA increased NT-3 levels in the spinal cord tissue surrounding the lesion site (unpublished data). These data suggest that EA treatment may stimulate NT-3 secretion from neuroglial cells and neurons in tissue adjacent to the lesion site. Both our previous study and the present study show the NT-3 level 2 weeks after spinal cord transection is higher than the level 4 weeks after transection, but the EA and MSCs+EA groups consistently maintained higher levels of NT-3 during this time compared to the Op-control or MSCs groups. Interestingly, in the present study, we found that EA combined with MSC transplantation significantly increased the quantity of NT-3 within the lesion site as compared to EA treatment alone. Our results also showed that some grafted MSCs were NT-3 immunopositive cells in the MSCs+EA group (data not shown). We propose that increased NT-3 content in the MSCs+EA treatment group is the result of a synergistic effect of EA treatment and MSC transplantation, as some studies have reported that transplanted MSCs can produce NT-3 [24] or stimulate neuroglial cells to produce neurotrophic factors [52] in the central nervous system. MSCs may also secrete cytokines and growth factors such as NGF, BDNF, and VEGF [23,53].

In this study, we found that the survival and differentiation of MSCs into neuron-like cells and oligodendrocytes were significantly increased in the MSCs+EA group, corresponding to a similar elevation in NT-3 and cAMP levels. This data suggests that EA promotes the survival and differentiation of grafted MSCs by elevating neurotrophic factors (such as NT-3) and the level of cAMP in injured spinal cord tissue. NT-3 is a significant member of the neurotrophic factors family and plays an important role during nervous system development, neuronal survival and differentiation, and neuronal repair via a signal transduction pathway [54]. In particular, NT-3 can induce both the survival and proliferation of oligodendrocytes by differential involvement of the transcription factor CREB [55,56]. In vivo studies of animal models have suggested that NT-3 may play an important role in regulating the quantity of oligodendrocytes and myelin regeneration following a CNS injury and demyelination [51,57]. In addition, some studies have demonstrated that cAMP provides a powerful survival signal for neurons [42,58].

However, several observations have raised questions regarding neuronal transdifferentiation of MSCs and suggested that the transdifferentiation was attributed to cell fusion with host cells. Several laboratories have proposed that MSCs fuse with the host cells, including the neurons, and acquire their phenotypes, which simulate their transdifferentiation into host cells [59-62]. While cell fusion could explain a good fraction of this apparent neural transdifferentiation of transplanted MSCs, there continues to be new evidence to suggest that, at least in some particular experimental paradigms, transdifferentiation does indeed occur [63-65]. Munoz-Elias et al. [64] transplanted adult rat bone marrow stromal cells into embryonic brains and observed that donor cells entered ventricular germinal zones, expressed neural progenitor traits, migrated to distant brain regions, and expressed site-specific neuronal proteins without cell fusion phenomena. Recently, Hokari et al. [66] found that bone marrow stromal cells might have the potential not only to differentiate into neurons, but also may fuse spontaneously with host neurons within 24 hr of cell-mixing coculture commencement. However, we never observed multiple nuclei originating from MSCs in the double-labeled cells during our study. Additionally, the NF150, β-tublin III, and 5-HT positive cells differentiated from grafted MSCs had few/no processes, which is not the typical morphology of host neurons. Furthermore, previous studies have suggested that this fusion is a rare event [59,60]. Thus, we believe MSC-derived neuron-phenotypic cells were mostly transdifferentiated from donor cells, although we could not absolutely rule out host cell fusion. In addition, we found that the MSCs+EA group had more double-labeled neuron-phenotypic cells from MSCs than the MSCs group. We are not clear whether EA promotes grafted MSCs to transdifferentiate or fuse with host cells, so we will further study the effect of EA on the fate of grafted MSCs in the future.

We also found that 5-HT and CGRP-positive immunostained axons were regenerated in or across the lesion site into the caudal spinal cord at different degrees, most prominently in the MSCs combined with EA treatment group. It is known that scar formation by glia proliferation, lack of tropic support and inhibitory molecules [67] are key factors that block axons from regenerating into the injured spinal cord. However, several studies indicate that EA treatment can increase the tissue cAMP level and the expression of neurotrophic factors, such as NT-3, BDNF, NGF, and GDNF [68,69]. Similarly, several studies [70,71] have demonstrated that MSCs also secrete a variety of growth factors and cytokines, which can promote axonal growth in vitro as well as in vivo [43,72,73]. In addition, Yang et al. reported that EA treatment can inhibit the reactive proliferation of astrocytes after spinal cord injury and prevent the formation of a glial scar [74]. Therefore, combining MSC transplantation and EA treatment may synergistically modify the hostile environment in the lesion site to promote axonal regrowth by increasing the amount of neurotrophic factors and the cAMP level and inhibiting glia scar formation.

The detection and behavioral analysis of spinal cord evoked potentials suggest that MSC transplantation combined with EA treatment efficiently improves neuronal function recovery. MSCs transdifferentiate into neurons and oligodendrocytes to replace the damaged or dead neural cells or repair myelin sheaths in the injured spinal cord, and increase the number of 5-HT-fibers passing through the lesion site into the caudal spinal tissue, which may be the morphological basis of the functional outcomes. However, whether or not these "neural cells" neurons were in fact functionally replaced damaged spinal cord neurons needs to be confirmed.

## Conclusion

In summary, our experiments have shown that MSC transplantation combined with EA treatment promotes MSC survival and differentiation into neural-like cells, stimulates axonal growth into the lesion/transplant site, and improves hindlimb locomotion. Increased NT-3 and cAMP levels in the injured/transplanted area may be the main molecular mechanism of the morphological and functional effects of the treatment. Our results suggest a new therapeutic potential of the combination of MSC transplantation with EA treatment to treat spinal cord injury patients.

## Authors' contributions

YSZ designed the study, contributed to the experiments, and drafted the manuscript. YD designed the study, contributed to all experiments and analysis of the data, and drafted the manuscript. YQ participated in the design of the study, conducted the surgery and behavioral examination of rats and analyzed the subsequent data. JWR carried out the electro-acupunture treatment on rats. YQZ contributed to the cell cultures and performed the spinal cord transections in some rats. WJL and YJZ also performed some of the spinal cord transections. YL contributed to the creation of the antibody protocol. HXD contributed to the study design and general concept, and drafted the manuscript. All authors read, commented, and approved the final manuscript.

## Supplementary Material

Additional file 1 **A sketch of the injured spinal cord to clarify the location of quantification (rostral, caudal or the lesion site)**. The quantification of 5-HT positive fibers was performed at three regions (0.3 mm rostral to the transection site, the transection site and 0.3 mm caudal to the transection site) which were delineated by 4 red lines. The CGRP-positive fibers were quantified only in the lesion site (outlined by the two middle red lines) at 200 × magnification. Click here for file

Additional file 2 **BrdU and Hoechst co-labeling images in vitro and in vivo**. A. The percentage of BrdU (red) and Hoechst (blue) co-nuclei (arrows) of MSCs was about 95% in vitro. B. Hoechst-labeled nuclei (blue, arrows) in the lesion site the MSCs+EA group. C. BrdU immunofluorescence showing BrdU-labeled nuclei (green, arrows) in the same field of A. D. image A and B showing BrdU and Hoechst co-nuclei of MSCs (blue-green, arrows). Scale bars: A = 40 μm, B, C, D = 20 μm. Click here for file
