# Supplementary material for: Electro-acupuncture promotes survival, differentiation of the bone marrow mesenchymal stem cells as well as functional recovery in the spinal cord-transected rats
Source: BMC Neurosci. 2009 Apr 20;10:35. doi: 10.1186/1471-2202-10-35 (PMC2679038; doi:10.1186/1471-2202-10-35)
Supplement: Additional file 1 — A sketch of the injured spinal cord to clarify the location of quantification (rostral, caudal or the lesion site). The quantification of 5-HT positive fibers was performed at three regions (0.3 mm rostral to the transection site, the transection site and 0.3 mm caudal to the transection site) which were delineated by 4 red lines. The CGRP-positive fibers were quantified only in the lesion site (outlined by the two middle red lines) at 200 × magnification. [file 1471-2202-10-35-S1.pdf]

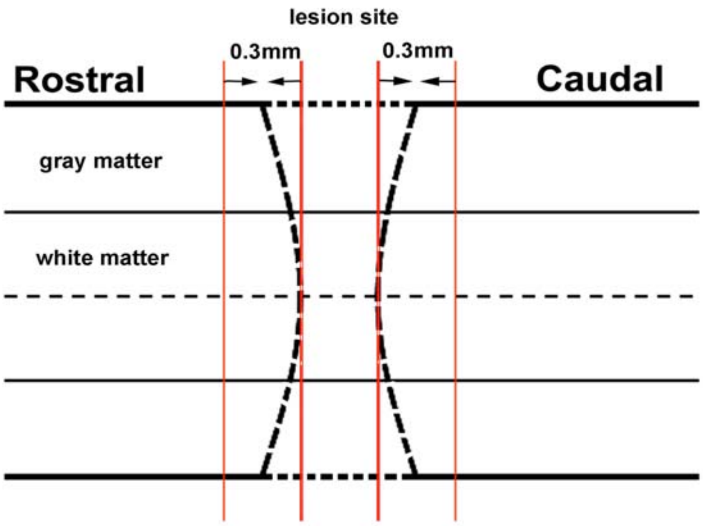

A sketch of the injured spinal cord to clarify the location of quantification (rostral, caudal or the lesion site).

The quantification of 5-HT positive fibers was performed at three regions (0.3mm rostral to the transection site, the transection site and 0.3mm caudal to the transection site) which were delineated by 4 red lines. The CGRP-positive fibers were quantified only in the lesion site (outlined by the two middle red lines) at 200× magnification.
