# Supplementary material for: Electro-acupuncture promotes survival, differentiation of the bone marrow mesenchymal stem cells as well as functional recovery in the spinal cord-transected rats
Source: BMC Neurosci. 2009 Apr 20;10:35. doi: 10.1186/1471-2202-10-35 (PMC2679038; doi:10.1186/1471-2202-10-35)
Supplement: Additional file 2 — BrdU and Hoechst co-labeling images in vitro and in vivo. A. The percentage of BrdU (red) and Hoechst (blue) co-nuclei (arrows) of MSCs was about 95% in vitro. B. Hoechst-labeled nuclei (blue, arrows) in the lesion site the MSCs+EA group. C. BrdU immunofluorescence showing BrdU-labeled nuclei (green, arrows) in the same field of A. D. image A and B showing BrdU and Hoechst co-nuclei of MSCs (blue-green, arrows). Scale bars: A = 40 μm, B, C, D = 20 μm. [file 1471-2202-10-35-S2.pdf]

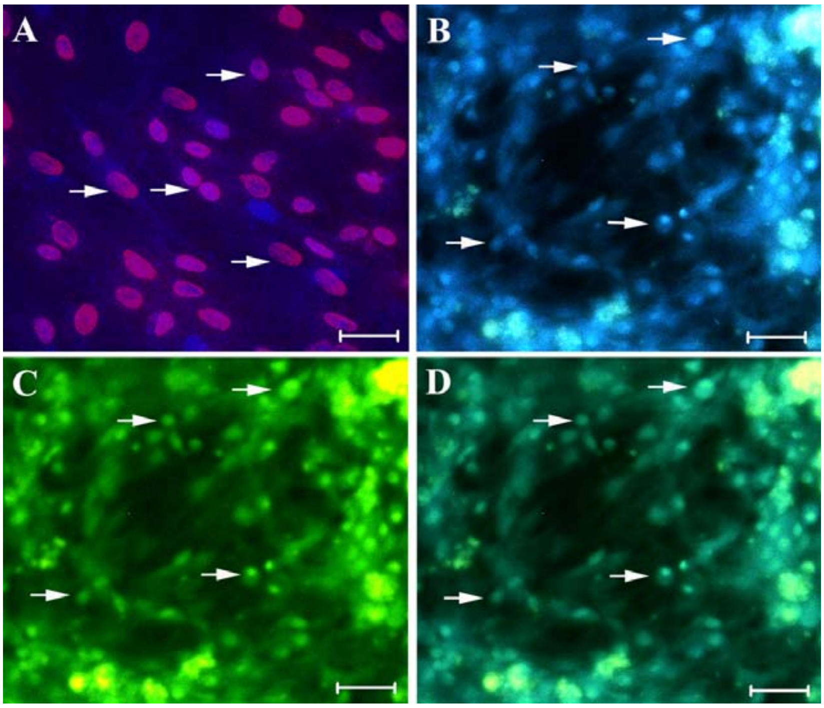

BrdU and Hoechst co-labeling images in vitro and in vivo.
A. The percentage of BrdU (red) and Hoechst (blue) co-nuclei (arrows) of MSCs was about 95% in vitro. **B.** Hoechst-labeled nuclei (blue, arrows) in the lesion site the MSCs+EA group. **C.** BrdU immunofluorescence showing BrdU-labeled nuclei (green, arrows) in the same field of **A**. **D.** image **A** and **B** showing BrdU and Hoechst co-nuclei of MSCs (blue-green, arrows). Scale bars: **A**=40μm, **B**, **C**, **D**=20μm
